# Supplementary material for: Master Regulators of Causal Networks in Intestinal- and Diffuse-Type Gastric Cancer and the Relation to the RNA Virus Infection Pathway
Source: Int J Mol Sci. 2024 Aug 13;25(16):8821. doi: 10.3390/ijms25168821 (PMC11354771; doi:10.3390/ijms25168821)
Supplement: Supplementary file 1 [file ijms-25-08821-s001.zip › figure caption.pdf]

Figure S1: Original image of Figure 3a The HDAC1-interacting network in diffuse-type GC, Figure S2: Original image of Figure 3b The HDAC1-interacting network in intestinal-type GC, Figure S3: Original image of Figure 4a The regulators of the HDAC1-regulated causal network with depth 3 in diffuse-type GC, Figure S4: Original image of Figure 4b The regulators of the HDAC1-regulated causal network with depth 3 in intestinal-type GC, Figure S5: Original image of Figure 6a The causal network (depth 3) of lenvatinib in diffuse-type GC, Figure S6: Original image of Figure 6b The causal network (depth 3) of lenvatinib in intestinal-type GC.
