# Supplementary figures and images for: Master Regulators of Causal Networks in Intestinal- and Diffuse-Type Gastric Cancer and the Relation to the RNA Virus Infection Pathway
Source: Int J Mol Sci. 2024 Aug 13;25(16):8821. doi: 10.3390/ijms25168821 (PMC11354771; doi:10.3390/ijms25168821)

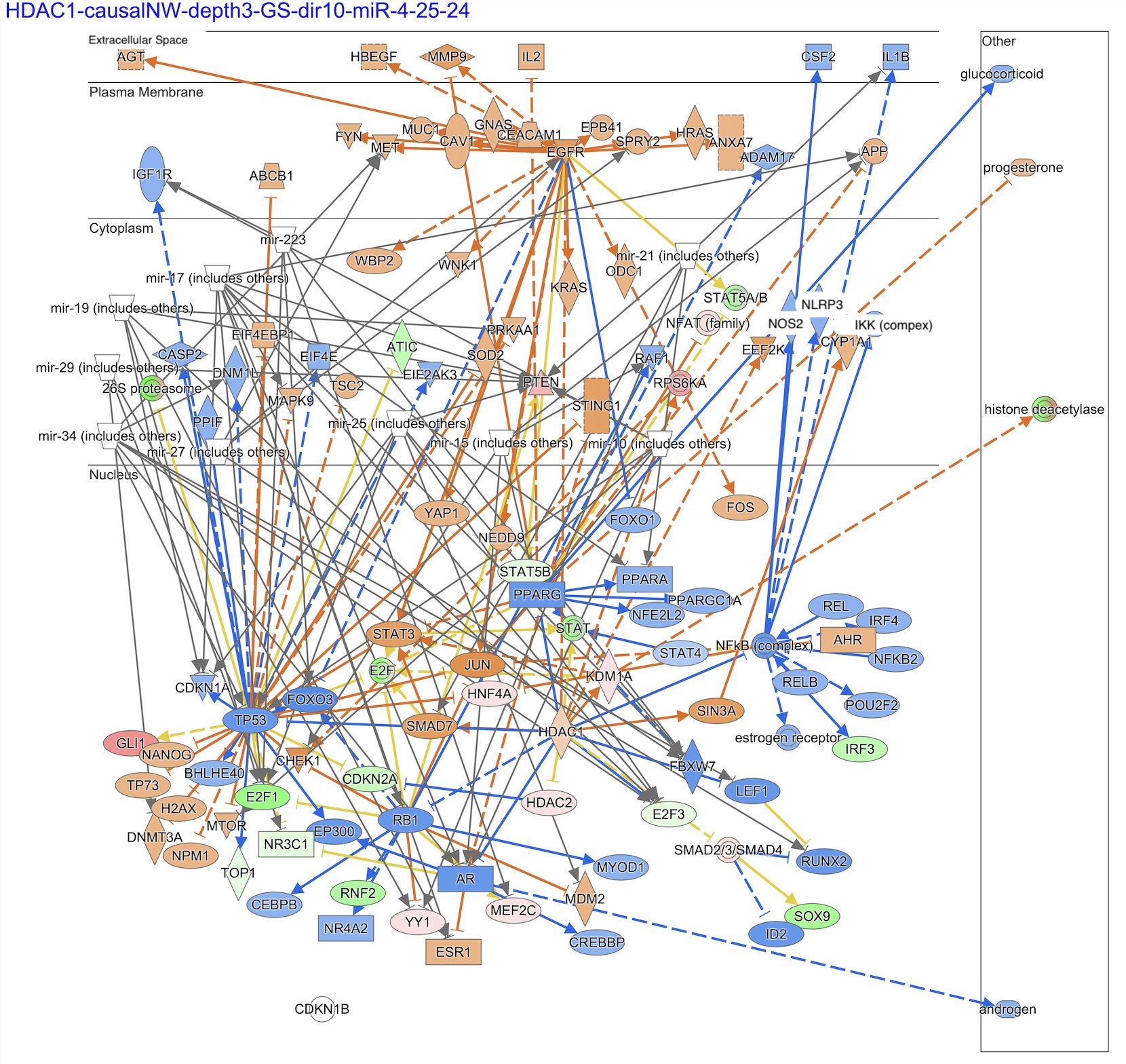

Supplement: Supplementary file 1 [file ijms-25-08821-s001.zip › FigS3-Fig4a.jpg]

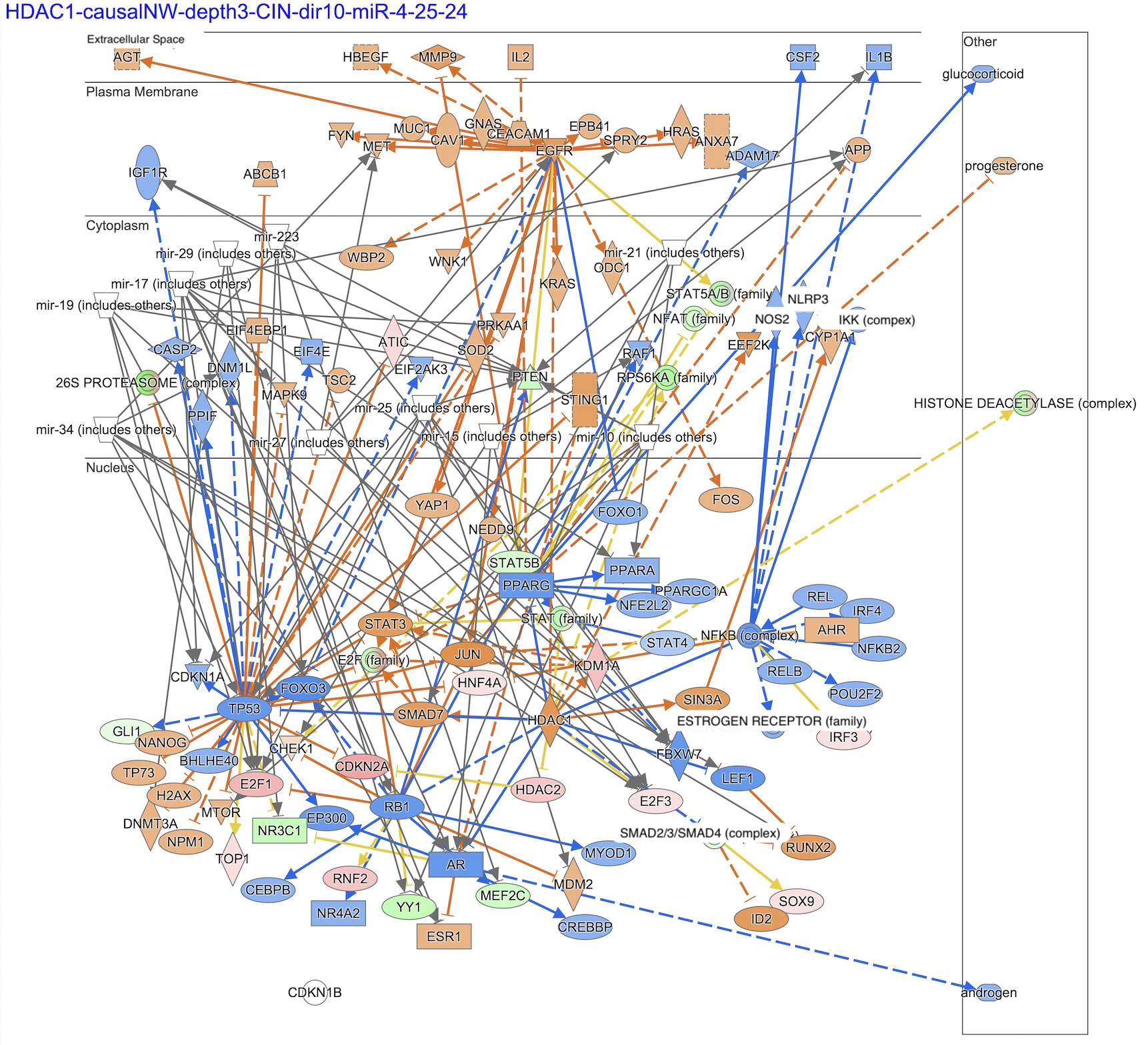

Supplement: Supplementary file 1 [file ijms-25-08821-s001.zip › FigS4-Fig4b.jpg]

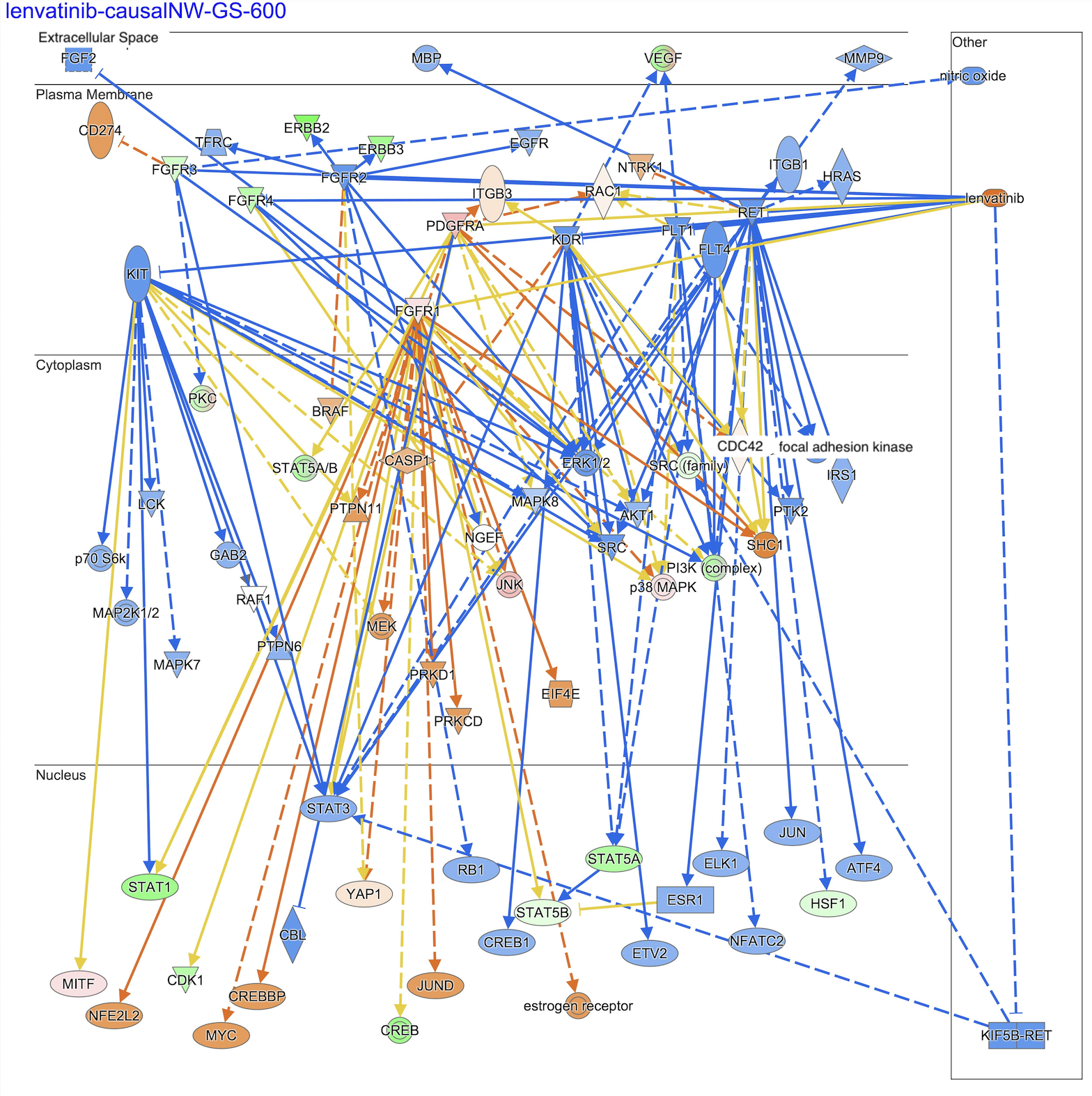

Supplement: Supplementary file 1 [file ijms-25-08821-s001.zip › FigS5-Fig6a.png]

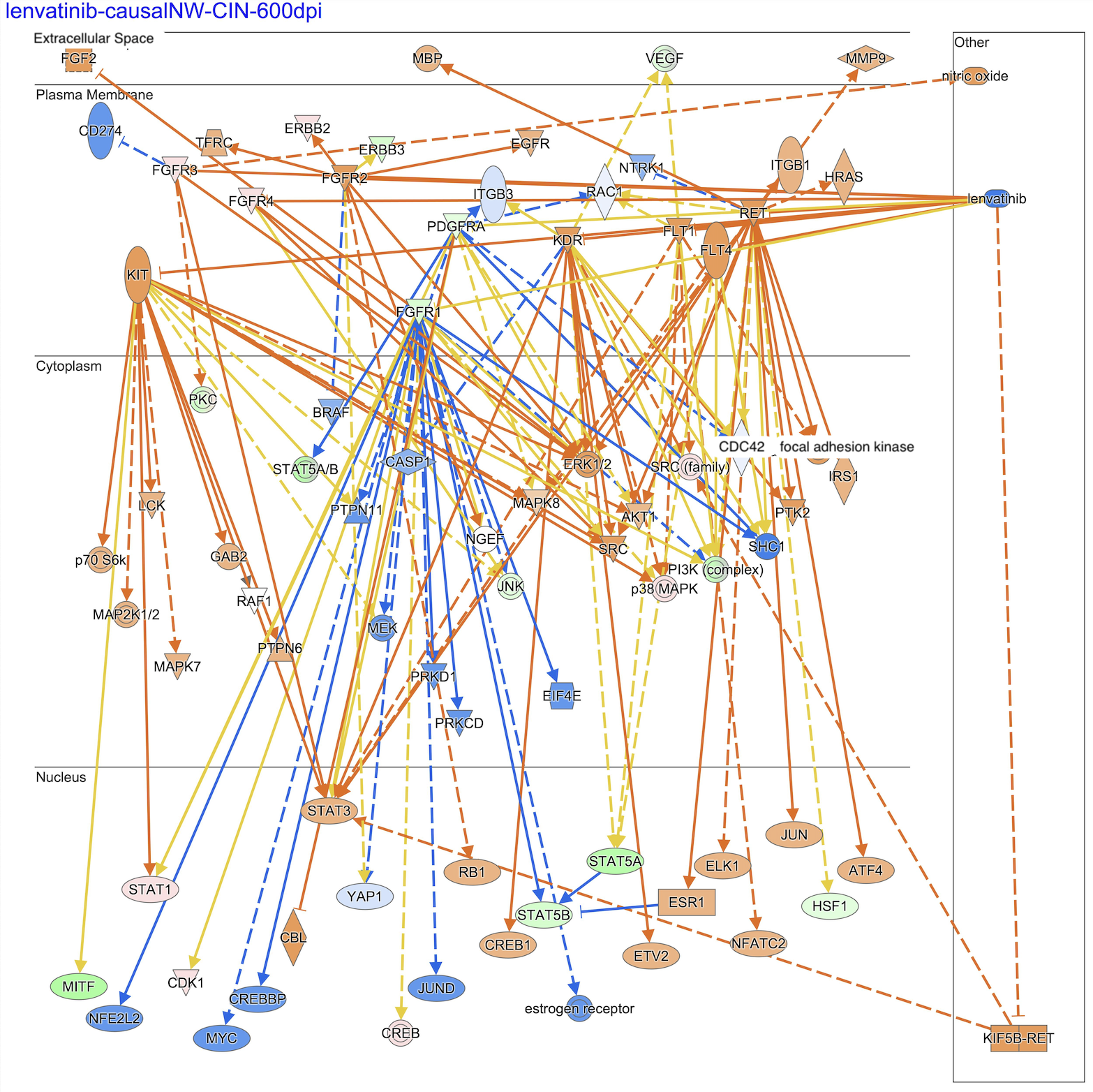

Supplement: Supplementary file 1 [file ijms-25-08821-s001.zip › FigS6-Fig6b.png]
